# Exposure to galactic cosmic radiation compromises DNA repair and increases the potential for oncogenic chromosomal rearrangement in bronchial epithelial cells

**DOI:** 10.1038/s41598-018-29350-5

**Published:** 2018-07-23

**Authors:** Z. Li, K. K. Jella, L. Jaafar, S. Li, S. Park, M. D. Story, H. Wang, Y. Wang, W. S. Dynan

**Affiliations:** 1 Department of Radiation Oncology, Emory University School of Medicine, 1365 Clifton Rd NE, Atlanta, GA 30322 USA; 2 Department of Biochemistry, Emory University School of Medicine, 1510 Clifton Rd NE, Atlanta, GA 30322 USA; 3 Winship Cancer Institute, Emory University School of Medicine, 1365 Clifton Rd NE, Atlanta, GA 30322 USA; 4 Department of Radiation Oncology, University of Texas Southwestern Medical Center, 6000 Harry Hines Blvd, Dallas, TX 75390 USA; 5 Department of Physics, Radiation Institute for Science and Engineering (RaISE), 100 University Dr, Prairie View A&M University, Prairie View, TX 77446 USA

## Abstract

Participants in deep space missions face protracted exposure to galactic cosmic radiation (GCR). In this setting, lung cancer is a significant component of the overall risk of radiation-exposure induced death. Here we investigate persistent effects of GCR exposure on DNA repair capacity in lung-derived epithelial cells, using an enzyme-stimulated chromosomal rearrangement as an endpoint. Replicate cell cultures were irradiated with energetic ^48^Ti ions (a GCR component) or reference γ-rays. After a six-day recovery, they were challenged by expression of a Cas9/sgRNA pair that creates double-strand breaks simultaneously in the EML4 and ALK loci, misjoining of which creates an EML4-ALK fusion oncogene. Misjoining was significantly elevated in ^48^Ti-irradiated populations, relative to the baseline rate in mock-irradiated controls. The effect was not seen in γ-ray irradiated populations exposed to equal or higher radiation doses. Sequence analysis of the EML4-ALK joints from ^48^Ti-irradiated cultures showed that they were far more likely to contain deletions, sometimes flanked by short microhomologies, than equivalent samples from mock-irradiated cultures, consistent with a shift toward error-prone alternative nonhomologous end joining repair. Results suggest a potential mechanism by which a persistent physiological effect of GCR exposure may increase lung cancer risk.

## Introduction

After a pause of nearly 50 years, human deep space missions will likely resume in the next decade. Galactic cosmic radiation (GCR) exposure poses unique health risks to participants in these missions^1–4^. The GCR dose rate in deep space, beyond the protection of the earth’s magnetic field, is significantly higher than in low earth orbit. Current technology permits only limited spacecraft shielding against these elevated GCR levels.

GCR is composed primarily of energetic protons, helium nuclei, and heavier nuclei^5^. The last of these, known as high charge and energy (HZE) particles, are of particular concern because they have high linear energy transfer (LET) values in tissue, leading to dense ionization and complex, difficult-to repair DNA damage along their core radiation tracks^6–10^. Perhaps because of this complex damage, HZE particles are significantly more effective than low-LET radiation, such as γ-rays, for induction of cancer in animal models^11–13^. Further support for the importance of complex damage comes from a study in a model system, where enzyme-induced fragmentation at a single locus was shown to compromise first-line DNA repair pathways^14^.

In addition to damaging DNA directly, HZE particle exposure evokes persistent physiological effects, including genome instability, changes in gene expression, and induction of oxidative stress and inflammation^15–23^, reviewed in^2,3^. Sometimes termed “non-targeted effects,” these are not limited to cells directly traversed by a radiation track, but can be manifested in their progeny and neighbours as well. At doses and dose rates prevalent in the spacecraft environment, non-targeted effects are believed to significantly increase the cancer risk attributable to targeted effects alone^24^, see also earlier reviews^25,26^. The mechanisms underlying non-targeted effects, particularly in the context of HZE particle exposure, remain incompletely understood.

Here we test a hypothesis that one of the persistent physiological effects of HZE particle exposure is to compromise the function of the DNA double-strand break (DSB) repair machinery, favouring the use of error-prone repair mechanisms in post-irradiated cells. In humans, three pathways contribute to DSB repair. Canonical nonhomologous end joining (c-NHEJ), the default pathway, is fast and relatively accurate (reviewed in^27,28^). Alternative NHEJ (alt-NHEJ), relies on a different ensemble (or “bricolage”) of repair proteins, typically involves resection of the broken ends, and has a relatively high likelihood of incorrectly joining the broken ends to create chromosomal rearrangements^29–31^. The third pathway, homology-directed repair, is important for maintenance of genome stability but is limited to dividing cells, which make up a small subset of total cells in most adult tissues.

An early study comparing radiation cytotoxicity in wild-type and c-NHEJ deficient cells suggested that exposure to HZE particle radiation inhibits the c-NHEJ pathway, but left open the question whether this is a targeted effect (for example, trapping of non-productive c-NHEJ complexes by DNA damage products) or a non-targeted effect^32^. More recently, we addressed the question of c-NHEJ capacity in post-irradiated cells using an experimental design that provides temporal separation between irradiation and repair of a fresh DSB introduced enzymatically after days or weeks of recovery. Reporter-bearing tumour cells were irradiated, allowed to recover, and challenged with I-SceI to induce recombinogenic breaks in transgene cassettes. Based on a fluorescence readout, misrepair is two to four-fold elevated in cells that were previously exposed to HZE particle radiation^33–35^. The effect persists for up to three weeks post-irradiation and occurs in both directly irradiated populations and in co-cultured, radiation-naïve bystanders, indicating that it is a non-targeted effect. The ability to extrapolate these observations to human carcinogenesis is limited, however, as they were performed in a tumour cell background, where regulation of repair may differ from that in normal cells, and the readout was based used an artificial reporter system, rather than endogenous, cancer-relevant genes.

We undertook the present study to validate the effect in a carcinogenesis-relevant model. We also performed sequence analysis of the novel DNA joints to gain insight into the underlying mechanism of the effect. We irradiated cells, allowed them to recover, and used Cas9/sgRNA technology to introduce recombinogenic breaks in the linked, endogenous EML4 and ALK loci^36^. Incorrect joining of the broken ends leads to a paracentric inversion and formation of an EML4-ALK oncogene, which is the single most common chromosomal rearrangement in spontaneous human lung cancers (reviewed in^37^). Experiments were performed using a derivative of a human bronchial epithelial cell line that has been widely studied as a model for lung carcinogenesis^38^. Results showed that cells with a history of HZE particle, but not γ-ray, exposure exhibited a significant increase in Cas9/sgRNA-stimulated EML4-ALK inversion frequency, relative to their mock-irradiated counterparts. The novel EML4-ALK joints formed in cells with a history of HZE irradiation also showed a high frequency of deletions, sometimes flanked by microhomologies. Results are consistent with a model in which exposure to HZE particle radiation fosters increased use of a resection-dependent, error-prone NHEJ pathway.

## Results

### Assay for EML4-ALK rearrangement

**Figure 1:**
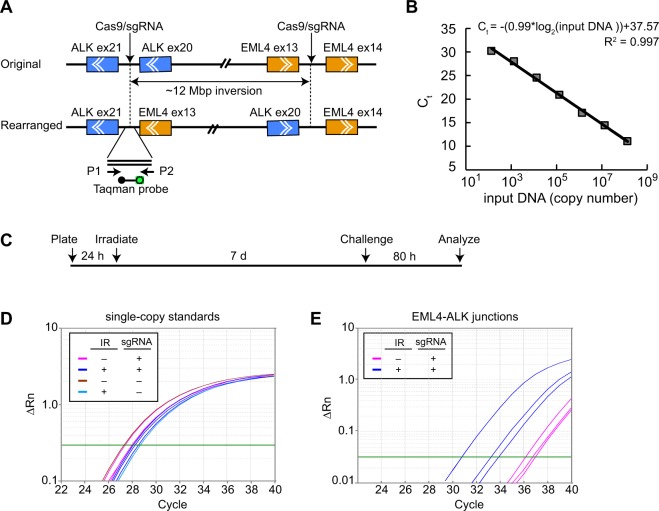
Experimental strategy and example data. (**A**) Schematic depicting sites of Cas9/sgRNA cleavage relative to ALK and EML4 genes. Chevrons depict direction of transcription. Cleavage sites are within introns. Exon (ex) numbers are shown. P1 and P2 are primers for Taqman PCR assays. Taqman hybridization probe is shown with fluorophore (green) and quencher (black) that separate upon hybridization to amplicon. (**B**) Reconstruction experiment showing linearity of Taqman qPCR assay. (**C**) Experimental timeline showing irradiation, recovery, Cas9/sgRNA challenge, and harvesting of DNA for analysis. (**D,E**) Example Taqman PCR data. Independent cultures were irradiated, challenged, and DNA collected for analysis. Amplification curves are shown for single-copy internal standard (RNase P, Panel D) or for EML4-ALK junctions (Panel E). Irradiation was performed at an LET of 108 keV/μm). Parallel cultures not challenged with Cas9/sgRNAs were included in the experiment in Panel E but showed no detectable amplification products after 40 cycles of amplification. Plots depict normalized fluorescence reporter values (ΔRn) as a function of cycle number. Green line denotes software-determined threshold for determination of amplification (C_t_) values.

Choi and Myerson described an approach using Cas9/sgRNA technology for induction of oncogenic rearrangements affecting endogenous genes^36^. A pair of Cas9/sgRNA nucleases are used to make separate incisions in the linked, endogenous ALK and EML4 loci. Misrepair, with inversion of an approximately 12 Mbp intervening sequence, creates an EML4-ALK fusion (Fig. 1A). The fusion protein is expressed at high levels and can be detected by immunofluorescence or flow cytometry using anti-ALK antibodies. In addition, the novel EML4-ALK junction can be detected by PCR using primers flanking the novel junctions. The original studies were performed in transformed, SV40 T-antigen expressing, HEK-293T cells. Fig. S1 shows replication of these results. Consistent with the original report, EML4-ALK joining was dependent on expression of both Cas9 and sgRNAs. In the HEK-293T model, the inversion event occurred with a frequency of up to 10.5% (Fig. S1).

For the main study of radiation effects on DSB repair in human bronchial epithelial cells, we adopted a sensitive and specific assay for qPCR detection of the novel EML4-ALK junctions using Taqman methodology. In a reconstruction experiment, the Taqman assay proved to be linear across a three order of magnitude range in input DNA concentration (Fig. 1B). Irradiation experiments used a human bronchial epithelial cell line, HBEC3-KT F25F, that was immortalized and transformed to anchorage-independent growth, but remains non-tumorigenic, as a model for precancerous lung cells. Replicate cultures were exposed to energetic ^48^Ti ions, allowed to recover for 7 d, and challenged by expression of the Cas9/sgRNA pair. After a further 80 h, genomic DNA was harvested for analysis.

Representative raw data from Taqman qPCR analyses of a single-copy RNase P gene standard and of EML4-ALK junctions are shown in Fig. 1D,E, respectively. For the single-copy standard, amplification curves were tightly clustered among replicates, reflecting good technical reproducibility for DNA isolation and amplification. For the EML4-ALK junctions, amplification curves showed a low, but detectible, occurrence in mock-irradiated cultures and a higher, albeit variable, frequency in post-irradiated cultures. The variability was typical and perhaps reflects the stochastic nature of non-targeted effects (see Discussion).

Comparison of the number of cycles required to reach the same normalized fluorescence reporter values (ΔRn) indicates that the EML4-ALK junctions require 6–12 additional cycles of amplification compared to the single-copy standard. Assuming equal efficiencies for the different primer-probe sets, the frequency of EML4-ALK fusion alleles in these cell populations thus ranged from about 0.025% (1/2^12^) in the mock-irradiated groups to 1.5% (1/2^6^) in the most responsive irradiated group.

### Quantitative analysis of dose and radiation quality effects

We performed a large-scale experiment to explore the frequency of misrepair in cell populations with different irradiation histories. The inhibitory effect of HZE particle radiation on c-NHEJ is thought to reflect levels of complex DNA damage products^32^, which are in turn a function of radiation dose and LET values, with higher LETs resulting in more complex damage at a given dose. We thus performed irradiations at three ^48^Ti doses (0.2, 0.5, and 1.0 Gy). By modulating the ^48^Ti beam energy, we were also able to perform irradiations at two LET values (108 and 200 keV/μm). Particle fluences and estimated mean hits per cell nucleus are shown in Table S1. Cells were allowed to recover for 7 d and challenged by expression of Cas9/sgRNAs. After 80 h, DNA was extracted and analysed as in Fig. 1. At these doses and energies, there was little or no effect of irradiation on cell viability at 48 h post-irradiation (Fig. S2A and S2B). Moreover, cell counts at the time of sub-culturing indicated that populations had undergone approximately one doubling (Fig. S2C), suggesting that the majority of the cells in the challenged population were progeny of the directly irradiated cells.

**Figure 2:**
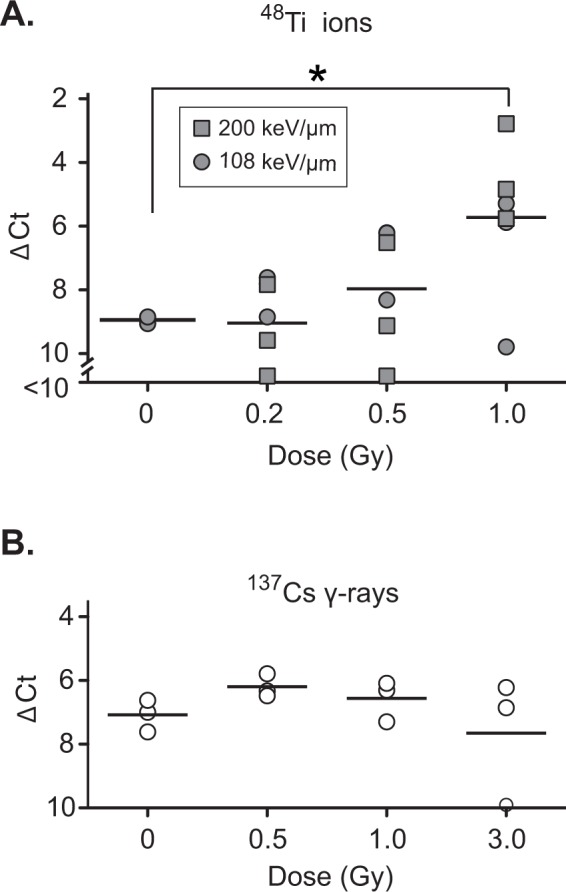
Effect of irradiation on the frequency of Cas9/sgRNA-induced EML4-ALK rearrangement. (**A**) Effect of 108 keV/μm or 200 keV/μm ^48^Ti on response to Cas9/sgRNA challenge. Triplicate cultures were irradiated as indicated, challenged by transduction with lentiviral Cas9/sgRNA vectors, and DNA was analysed by Taqman qPCR using RNase P as an internal standard. Significance was evaluated by ANOVA followed by 2-sided Dunnett t-tests *P < 0.05. (**B**) Same as panel A but irradiation with ^137^Cs γ-rays. Differences were non-significant. Experiments in panel A and B were performed contemporaneously at the NASA Space Radiation Laboratory or a ^137^Cs irradiator, both of which are located within the Brookhaven National Laboratory.

Results showed a marked increase in the frequency of Cas9/sgRNA-stimulated inversions with increasing radiation dose (Fig. 2A). As there was no significant difference in results, values obtained with the 108 and 200 keV/μm ion beams at each dose were pooled for statistical analysis. The increase in inversion frequency in the 1.0 Gy group, relative to the mock-irradiated control group, was slightly more than 3 ΔCt units, corresponding to a difference in allele frequency of 9-fold, and was statistically significant at the p < 0.05 level. The 0.5 Gy group showed a trend toward increased in frequency of rearranged alleles, although the differences with the mock-irradiated group was not statistical significant. We also investigated the effect of reference ^137^Cs γ-rays, a form of low-LET radiation (0.67 keV/μm). There was no effect at doses of up to 3.0 Gy (Fig. 2B).

We performed an independent, smaller-scale experiment during a separate beam line campaign. Results are shown in Fig. S3. Here, duplicate cultures were irradiated at two ^48^Ti doses (0.3 and 1.0 Gy) at a single LET value (108 keV/μm). There was a significant increase in Cas9/sgRNA-stimulated inversion frequency at the 1.0 Gy dose, relative to mock-irradiated control cultures (Fig. S3). The ability to replicate the key finding in two beam line campaigns, approximately six months apart, gives confidence in the robustness of the results.

### Misrepair junction characterization

**Figure 3:**
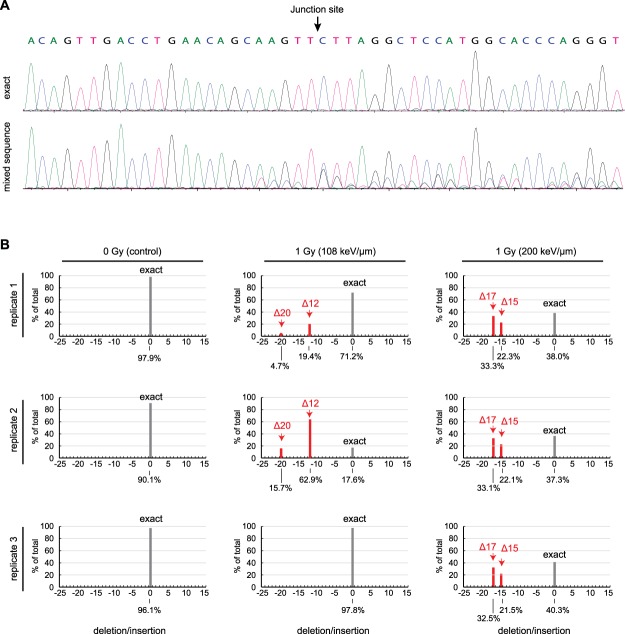
TIDE analysis of Cas9/sgRNA-stimulated EML4-ALK junctions. (**A**) Representative primary sequence data. Top panel, amplification of cloned DNA representing exact joining of Cas9/sgRNA-generated blunt ends. Bottom panel, amplification of pooled genomic DNA from a flask of HBEC-3KT F25F cells irradiated with 1.0 Gy of 108 keV/μm ^48^Ti ions and subjected to Cas9/sgRNA challenge. (**B**) TIDE analyses of DNA from multiple independent cell populations. Samples are from the same experiment as in Fig. 2A. Red bars denote recurrent deletions; grey bars denote exact joining. Only peaks scored as significant are shown. Contribution of each species to the total is indicated (%). Percentages sum to less than 100 because non-significant peaks are not scored or shown.

To gain further insight into the underlying mechanisms associated with DSB misjoining, we analysed the DNA sequences of the novel EML4-ALK junctions. Genomic DNA from irradiated or mock-irradiated control cell populations was extracted, and the junctional sequences were amplified by nested PCR. The mixed reaction products were subjected to Sanger sequencing and analysed at the population level using the Tracking of Indels by Decomposition (TIDE) method^39^. Examples of electropherograms representing an exact EML4-ALK joint, versus the mixed products obtained by PCR amplification of a genomic DNA sample, are shown in the top and bottom panels of Fig. 3A, respectively. For the mixed products, the sequence is unambiguous in the primer-proximal region (left side) but becomes ambiguous 5 nt prior to the junction site, indicating the presence heterogeneous insertions or deletions in the genomic DNA population.

The TIDE algorithm analyses the electropherograms to identify the major indel species and estimate their frequency. In the mock-irradiated cultures, >90% of Cas9/sgRNA-stimulated EML4-ALK joints arose from exact joining of the blunt end cleavage products. By contrast, in the irradiated cultures, a significant fraction of the junctions harboured deletions (Fig. 3B). Two recurrent deletions (∆12 and ∆20) dominated the indel spectrum for cells exposed to ^48^Ti ions at an LET of 108 keV/μm, and another two (∆15 and ∆17) dominated the spectrum for cells exposed to ^48^Ti ions at an LET of 200 keV/μm.

**Figure 4:**
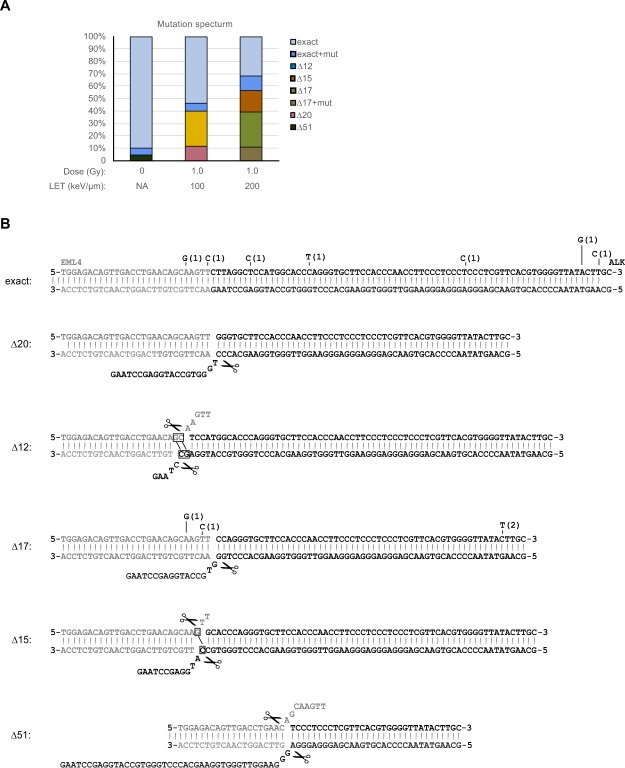
Sequence analysis of cloned junction sequence DNA. (**A**) Summary plot showing percentages of clones of different types recovered from different treatment groups. Note concordance with TIDE analysis in Fig. 3B. (**B**) Proposed origin of sequence deletions. Top sequence shows exact joining, other sequences show recurrent deletions. Deletions are assumed to occur by 3′ resection, followed by resection of unpaired sequence flaps (denoted by scissors icon). Microhomologies are boxed. Some clones contained point mutations as shown. No individual clone had more than one point mutation, and no mutation was recovered more than once in a given deletion context.

Individual DNAs were cloned from the amplified mixtures and sequenced, with results summarized in Fig. 4A. In the mock-irradiated populations, 18/19 clones (almost 95%) exhibited exact joining of the Cas9-cleaved ends. The clones were mostly free of point mutations, although there was one instance of a point mutation elsewhere in the sequenced region. Only one clone exhibiting a deletion (Δ51). By contrast, many or most of the clones from the irradiated populations harboured deletions, consistent with the TIDE predictions and indicative of joining by a resection-dependent repair mechanism. Figure 4B shows sequences of individual clones. Mutants ∆20 and ∆17 harboured unidirectional deletions extending into the ALK gene (Fig. 4B). Mutants ∆12 and ∆15 harboured bidirectional deletions extending into both the EML4 and ALK genes. The bidirectional deletions were flanked in both cases by 1–2 bp of microhomology (denoted by boxes), a hallmark of resection-mediated joining. The figure also illustrates the proposed mechanism of deletion formation, which involves limited resection to expose short 3′ tails, apposition or hybridization of DNA ends, and trimming of 3′ flap sequences.

We noted that some deletions were present exclusively in the 108 keV/μm populations and others exclusively in the 200 keV/μm populations. At this time, we do not have a hypothesis to explain why the pattern of recurrent deletions should be LET dependent, and we cannot rule out the possibility that it occurred by chance.

Some of the sequenced regions (11/86) contained point mutations, primarily AT > GC transitions. There was no significant association between the frequency of point mutations and the treatment group or the concurrent presence of deletions. There was also no significant enrichment proximal to the sequence junctions. Flanking point mutations are not typical products of Cas9/sgRNA mutagenesis, and we cannot exclude the possibility that the ones seen here are PCR artefacts.

## Discussion

Here we show that a prior history of energetic heavy ion exposure compromised the fidelity of DSB repair in a human lung-derived cell line. Specifically, the frequency of incorrect joining of two enzymatically-induced DSBs was significantly higher in cell populations that were previously exposed to ^48^Ti ions, a GCR component. In addition, sequencing of the novel junctions arising in the post-irradiated cells revealed the presence of recurrent, 12–20 nt deletions. In the most severely affected population (1.0 Gy, 200 keV/μm), almost 70% of the novel joints harboured deletions, far more than the 5% in the mock-irradiated control population.

Two different repair pathways, c-NHEJ and alt-NHEJ, contribute to rejoining of radiation-induced DSBs in mammalian cells. Although alt-NHEJ was initially considered to be the main source of misjoining to create chromosomal rearrangements, more recent work has shown that c-NHEJ can also promote DNA misjoining, particularly under conditions of ATM kinase deficiency^40,41^. However, even in the setting of misjoining, c-NHEJ typically generates indels of only a few bp at sites of rejoining, whereas alt-NHEJ generates larger deletions. Although further studies with mutant cells will be needed to be certain, the prevalence of >10 nt deletions in the irradiated samples is suggestive of alt-NHEJ than c-NHEJ mediated joining.

Alt-NHEJ was initially described as a backup pathway in cells or cell extracts that were deficient for c-NHEJ, which is the normal default pathway for end-joining in mammalian cells^42,43^. Several factors influence the regulation of alt-NHEJ. One of these is position in the cell cycle, with alt-NHEJ up-regulated in G2 phase^44^. We did not measure cell cycle distribution in the present study, although in a previous study using a different cell line we found only a slight overrepresentation of G2/M cells at 7d post-irradiation (18.00 ± 1.39% for 1.0 Gy vs. 15.53 ± 0.23% for non-irradiated controls)^33^, which would be too small to account for observed effects on repair pathway utilization. Interestingly, low-LET radiation stimulates alt-NHEJ in a transient, plasmid-based assay by stimulating phosphorylation of XRCC1^45^. Our attempts to detect changes in XRCC1 phosphorylation at 6 d post-irradiation have been unsuccessful. We note also a report that although XRCC1 reinforces the role of DNA ligase III in chromosomal translocations mediated by alt-NHEJ, it is not essential^46^.

An alternative explanation for an increased prevalence of alt-NHEJ-mediated joining is inhibition of c-NHEJ. A recent study showed that induction of even a small number of DNA fragments significantly affects repair pathway utilization^14^. An earlier study suggested that small DNA fragments, generated as direct products of high-LET radiation, bind to and inhibit the function of Ku protein^32^. If these were sufficiently persistent, they could influence results of a challenge initiated at 7 d post-irradiation. We are intrigued by a recent report that exposure to high-LET radiation generates small DNA fragments by a secondary mechanism involving resection by the MRE11 repair nuclease^47^. These engage the cytoplasmic cGAS receptor and trigger a STING-dependent pro-inflammatory response. We note that Ku protein, a key factor in the c-NHEJ pathway, is exquisitely sensitive to oxidative stress^48–50^. Inflammation and consequent changes in cellular redox status could thus provide an additional molecular basis for inhibition of c-NHEJ in post-irradiated cells.

In prior work using reporter lines, we demonstrated that co-culturing with HZE particle-irradiated cells increases misjoining of I-SceI induced DSBs in non-irradiated bystander cells^35^. Attempts to extend this finding to the epithelial cell model used here were unsuccessful. It could be that the increased use of error-prone pathways is a cell-autonomous effect in the HBEC3-KT F25F model, or alternatively that the magnitude of the effect in bystander cells was below the threshold for detection.

We noted considerable variability in the response of replicate cultures to the same doses of HZE particle radiation. While we cannot rule out the possibility of technical issues, the levels of the RNase P internal standard detected by qPCR were highly reproducible, and it was only the levels of EML4-ALK joints that were variable. We speculate that the variability might be biological in origin. One hypothesis is that there is a small, and thus variable, number of cells in each flask that drive the observed phenotype. Perhaps, these correspond to cells with persistent unrepaired damage. The presence of low-abundance driver cells is a subject of ongoing interest.

In space, organisms are subjected to a mixed GCR field. The fluence of low-LET ^1^H or ^4^He ions is far greater than that of heavier species^5^. There is considerable interest in how characteristic damages inflicted by different ion species interact to modify overall risk. The design of the present experiments, where cells were exposed to heavy ion irradiation to create complex DSBs, then later challenged by induction of simple DSBs, emulates a situation where traversal of a cell by a high-LET particle is followed by one or more encounters with low-LET ions, which create simple DSBs distributed throughout the genome. The observation that HZE particle exposure affects capacity for accurate repair of subsequent DSBs provides insight into how persistent physiological effects of heavy ion radiation might amplify cancer risk in the space environment.

An important caveat for beam line studies is that they are conducted using doses and dose rates that are higher than anticipated human exposures for an exploration-class mission^51^. It may be that different biological response mechanisms dominate in different dose regimes. The mechanisms considered here may not dominate or occur in space. The development of more sensitive biological models, and of ground-based facilities for low dose, mixed field, and protracted exposures to GCR components, remain as important research challenges.

## Methods

### Cell culture

HEK-293FT cells (Life Technologies, Carlsbad, CA) were grown in Dulbecco’s Modified Eagle Medium with 10% foetal bovine serum, 2 mM L-glutamine, 100 U/ml penicillin and 100 μg/ml streptomycin. HBEC3-KT F25F cells, which were derived from the parental HBEC3-KT line^38^ by selection for anchorage-independent growth following exposure to 0.25 Gy of ^56^Fe particle radiation, were grown in Keratinocyte-SFM medium supplemented with 50 µg/mL bovine pituitary extract and 5 ng/mL epidermal growth factor (Gibco) at 37 °C in 5% CO_2_.

### CRISPR/Cas9 expression plasmid construction and lentivirus production

Single guide RNA (sgRNA) sequences for EML4 (GACCTGAACAGCAAGTTTGT) and ALK (GGCCTTGCTGAAACTTCCTT) gene were previously described^36^. For the main experiments Cas9/sgRNA expression constructs were introduced by lentiviral transduction. EML4 and ALK sgRNAs were inserted, separately, into plentiCRISPR v2 (Addgene plasmid # 52961, ref.^52^) to construct plentiCRISPR v2-EML4 and plentiCRISPR v2-ALK respectively. Co-transfection of HEK-293FT cells was performed in 10 cm dishes using polyethylenimine (PEI) as described^34,53^. Each dish received using 13.6 μg of sgRNA plasmid, 3.4 μg of pMD2.G (Addgene plasmid # 12259), which encodes amphitrophic vesicular stomatitis virus (VSV) G coat protein, and 6.8 μg of psPAX2 (Addgene plasmid #12260), which encodes other proteins required for lentiviral packaging. Supernatants were collected at 48 h post-transfection and cleared by centrifugation for 10 min at 800 *g* at 4 °C. Supernatant was collected, and virus yield was estimated using Lenti-X GoStix (Clontech Laboratories, Inc., Mountain View, CA). Suspensions containing >5 × 10^5^ infectious units/ml were aliquoted and stored at −80 °C. For experiments in which the challenge was performed using a Cas9/sgRNA expression vector (Figs S1, S3), the EML4 and ALK sgRNAs were cloned downstream of separate U6 promoters in the pX330-U6-Chimeric_BB-CBh-hSpCas9 (Addgene # 42230, ref.^54^) to obtain pX330-EML4-ALK.

### Irradiation

HZE particle irradiations were performed at the NASA Space Radiation Laboratory (Brookhaven National Laboratory, Upton, NY) at a dose rate of 0.2 to 1.0 Gy/min in T-25 flasks positioned orthogonally to the beam. Beam energies for ^48^Ti ions were 1000 MeV/u (LET = 108 keV/μm) or 230 MeV/u (LET = 200 keV/μm). LET values, particle fluences, and mean hits per cell nucleus were estimated using the Galactic Cosmic Radiation Event-based Risk Model code (GERMcode v1.1 2000)^55^. Reference ^137^Cs γ-ray irradiation was performed at Brookhaven National Laboratory using a J.L. Sheppard & Associates MK I-68A irradiator at a dose rate of 1.5 Gy/min. Replicate cultures were maintained in separate flasks for all procedures from irradiation through harvest and analysis. Cultures were incubated for 6 d post-irradiation and equal numbers from each flask were subcultured into fresh T-25 flasks.

### Cas9/sgRNA challenge

The Cas9/sgRNA challenge was performed at 7 d post-irradiation (1 d after subculture.) For the main experiment in Fig. 2, flasks were washed with PBS and 1.5 ml aliquots of each of the two lentiviral Cas9/sgRNA vectors were added. After overnight incubation, virus stocks were removed and replaced with fresh culture medium. For the experiment in Fig. S1, the pX330-EML4-ALK plasmid vector was transfected using Lipofectamine 3000, and for the experiment in Fig. S3, the same vector was introduced by nucleofection using a Basic Epithelial Cell Nucleofector Kit (Lonza, #VPI-1005) and program T-13 on a Nucleofector 2b device (Lonza). In all instances, cells were harvested for analysis at 80 h following introduction of the Cas9/sgRNA vectors.

### PCR analysis of misrepair

Genomic DNA was isolated using a NucleoSpin Tissue kit (Clontech). For Taqman qPCR, reactions (20 μl) contained Taqman Fast Advanced Master Mix (Applied Biosystems), primers P1, d(CAGTTGTGTTGTTCAATTTTTAAGGT) and P2, d(CTGTGTTGCAAGTATAACCCC), and Taqman probe, d(CTTCCCTCCCTCCCTCGTTC). A single copy gene RNase P was amplified as an internal standard by the TaqMan Copy Number Reference Assay (Life Technologies). Cycling parameters were 50 °C for 2 min, 95 °C for 20 s, then 40 cycles of 95 °C for 1 s and 60 °C for 20 s. Data were processed using StepOne Software V2.2.2 (Life Technologies) and analysed using the ΔCt method^56^. Detection of EML4-ALK products in Fig. S2 was performed by an alternative method using SYBR Green qPCR. Reactions (20 μl) contained SYBR Select PCR Master Mix (Applied Biosystems) and primers P3, d(ATGTAAGTGGAGACAGTTGACC) and P4, d(GAAGGTTGGGTGGAAGCA). A sequence on intron 1 of ALK gene was amplified as an internal standard, using primer P5, d(AAGAGACTGGAGAGGGAACA) and P6, d(GATCGGCTCCTATGCAAATCT). Amplification was performed with cycling parameters of 50 °C for 2 min, 95 °C for 2 min, then 40 cycles of 95 °C for 15 s and 60 °C for 1 min. The endpoint PCR in Fig. S1 was performed using primers P7, d(GTCCTCCCTCTCGTGGTAAC) and P8, d(GCCCTTGAAGCACTACACAG) and LA Taq polymerase (Clontech) reaction with cycling parameters of 94 °C for 1 min, 35 cycles of 98 °C for 10 s, 56 °C for 1 min, 72 °C for 1 min, then 10 min at 72 °C.

#### Immunostaining

Cells for immunofluorescence were fixed with 2% paraformaldehyde for 10 min, washed, permeabilized with 0.5% Triton X-100 for 15 min, blocked with 10% goat serum for 1 h, stained with anti-ALK (Cell Signaling Technology, #3633 P, 1:200) and anti-FLAG-Cas9 (GeneTex Inc. #GTX80656, 1:200) antibodies for 1 h, then with Alexa Fluor 488-conjugated goat anti-rabbit (Life Technologies, #A11008, 1:500) and Alexa Fluor 594-conjugated goat anti-mouse (Life Technologies, #A11005, 1:500) antibodies for 1 h, and counterstained with 4′,6-diamidino-2-phenylindole (DAPI). Images were collected using a Deltavision microscope and analysed using Softworx software. Cells for FACS analysis were harvested, fixed with 4% paraformaldehyde for 10 min, permeabilized in 90% methanol for 30 min on ice. Cells were blocked and stained with anti-ALK (1:400) and anti-FLAG-Cas9 (1:200) antibodies as above, then with secondary antibodies as above. Cells were resuspended in PBS and analysed using a BD LSR II flow cytometer and FlowJo X software (FlowJo, LLC).

### Sequence analysis of novel EML4-ALK junctions

Novel EML4-ALK fusion junctions were examined using a two-round nested PCR approach. Genomic DNA (1 µg) was amplified in the first round PCR using primers P9 d(CCTTCAGGCTACTCTTGTTAGTT) and P10 d(TCACTGATGGAGGAGGTCTT), in 25 μl reactions with LA Taq polymerase (Clontech) and Mg^++^-containing reaction buffer. Amplification was with cycling parameters of 94 °C for 1 min, then 27 cycles of 98 °C for 10 s, 55 °C for 1 min, 72 °C for 1 min, then 10 min at 72 °C. 1.5 µl of amplification product was add to the second round PCR using primers P11 d(TGACCATGCACAGGGAAATAA) and P12 d(GTTAGTCTGGTTCCTCCAAGAAG), in 25 μl reactions with LA Taq polymerase (Clontech) and Mg^++^-containing reaction buffer. Amplification was with cycling parameters of 94 °C for 1 min, then 30 cycles of 98 °C for 10 s, 52.5 °C for 1 min, 72 °C for 1 min, then 10 min at 72 °C.

Total PCR amplicons from each sample were sequenced using primer P11 d(TGACCATGCACAGGGAAATAA). The raw trace files were then analysed using the TIDE (Tracking of Indels by Decomposition) web tool^39^. Individual EML4-ALK fusion junction sequences were detected by cloning single PCR amplicon into a pCR 2.1 vector, using an Original TA cloning Kit (Invitrogen).

### Data availability

The datasets generated during and/or analysed during the current study are available from the corresponding author on reasonable request.

## Electronic supplementary material

Supplementary information
