# Supplementary material for: Exposure to galactic cosmic radiation compromises DNA repair and increases the potential for oncogenic chromosomal rearrangement in bronchial epithelial cells
Source: Sci Rep. 2018 Jul 23;8:11038. doi: 10.1038/s41598-018-29350-5 (PMC6056477; doi:10.1038/s41598-018-29350-5)
Supplement: Supplementary file 1 — Supplementary information [file 41598_2018_29350_MOESM1_ESM.pdf]

SUPPLEMENTARY INFORMATION

Exposure to galactic cosmic radiation compromises DNA repair and increases the potential for oncogenic chromosomal rearrangement in bronchial epithelial cells

Z. Li[1], K.K. Jella[1], L. Jaafar[1], S. Li[1,2], S. Park[4], M. D. Story[4], H. Wang[5], Y. Wang[1,3], W. S. Dynan[1,2,3,*]

[1]Department of Radiation Oncology, Emory University School of Medicine, 1365 Clifton Rd NE, Atlanta, GA, USA 30322

[2]Department of Biochemistry, Emory University School of Medicine, 1510 Clifton Rd NE, Atlanta, GA, USA 30322

[3]Winship Cancer Institute, Emory University School of Medicine, 1365 Clifton Rd NE, Atlanta, GA, USA 30322

[4]Department of Radiation Oncology, University of Texas Southwestern Medical Center, 6000 Harry Hines Blvd, Dallas, TX, USA 75390

[5]Department of Physics, Radiation Institute for Science and Engineering (RaISE), 100 University Dr, Prairie View A&M University, Prairie View, TX, USA 77446

*Corresponding author.

Mailing address: Department of Biochemistry, Emory University School of Medicine, 4121 Rollins Research Center, 1510 Clifton Rd. NE, Atlanta, GA 30322, Mailstop: 1940-001-1AE (SOM: Biochemistry)

Tel: +1-404-727 4104; Email: wdynan@emory.edu (W.S. Dynan)

SUPPLEMENTARY TABLE

Table S1

Energy (MeV/u)	Dose (Gy)	LET (keV/µm)	Fluence ($1/\mu m^2$)	Mean hits/ nucleus
1000	0.2	108	0.0116	1.16
1000	0.5	108	0.0290	2.90
1000	1.0	108	0.0580	5.80
230	0.2	200	0.00623	0.62
230	0.5	200	0.0156	1.56
230	1.0	200	0.0312	3.12

LET, fluence, and mean hits/nucleus were estimated using the Galactic Cosmic Radiation Event-based Risk Model code (GERMcode v1.1 2000). The calculation assumed that the area of a HBEC3-KT F25F cell nucleus is 100 μm^2 (i.e., a diameter of approximately 11 µm).

SUPPLEMENTARY FIGURES

Figure S1.

A. Immunofluorescence images showing increased expression of ALK in cells harbouring the EML4-ALK fusion. HEK 293FT cells were transfected with vectors expressing Cas9 only or Cas9 with sgRNAs specific for EML4 and ALK, as indicated. Images show staining with anti-Cas9 (red), anti-ALK (green) or DAPI (blue). Arrows denote dual-positive, Cas9 and ALK-expressing cells. Scale bar, 50 µm. Images were deconvolved and projections are shown. B. FACS analysis of cells transfected as in A. Percentages of Cas9 single positive (Q1) and Cas9, ALK-dual-positive cells (Q2) are indicated. C. PCR amplification scheme to detect EML4-ALK junction. D. PCR results for cells transfected with indicated Cas9 or Cas9/sgRNA vectors. All lanes are from same gel. Refer to Fig. S4 for uncropped image. Horizontal line is a printer artefact.

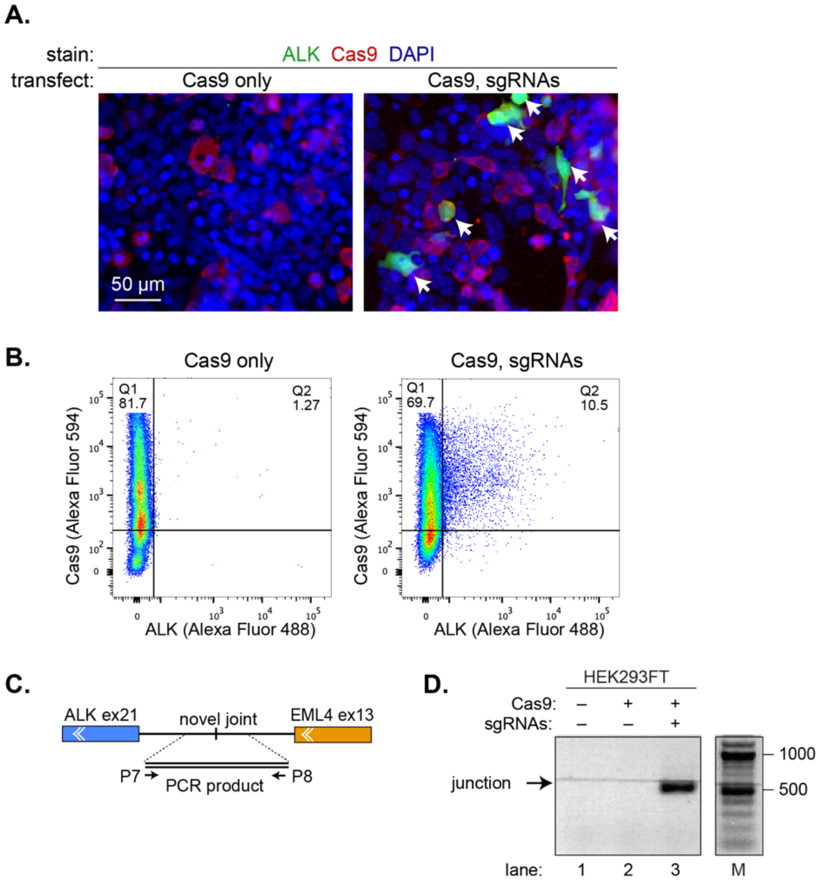

Figure S2. A, B. MTT viability assays performed 48 h following irradiation at indicated doses. Assays were performed using an MTT Cell Growth Assay Kit (EMD Millipore, #CT02). C. Cell growth. For Cas9/sgRNA assays, 8 X 10^5 cells were seeded in T-25 flasks. Cells were irradiated the following day, then incubated 6 d and equal numbers from each group were subcultured into fresh T-25 flasks for Cas9/sgRNA challenge. As a guide to subculturing, cell numbers for one of the three replicates were determined and are plotted here. Dashed line indicates number of cells seeded originally.

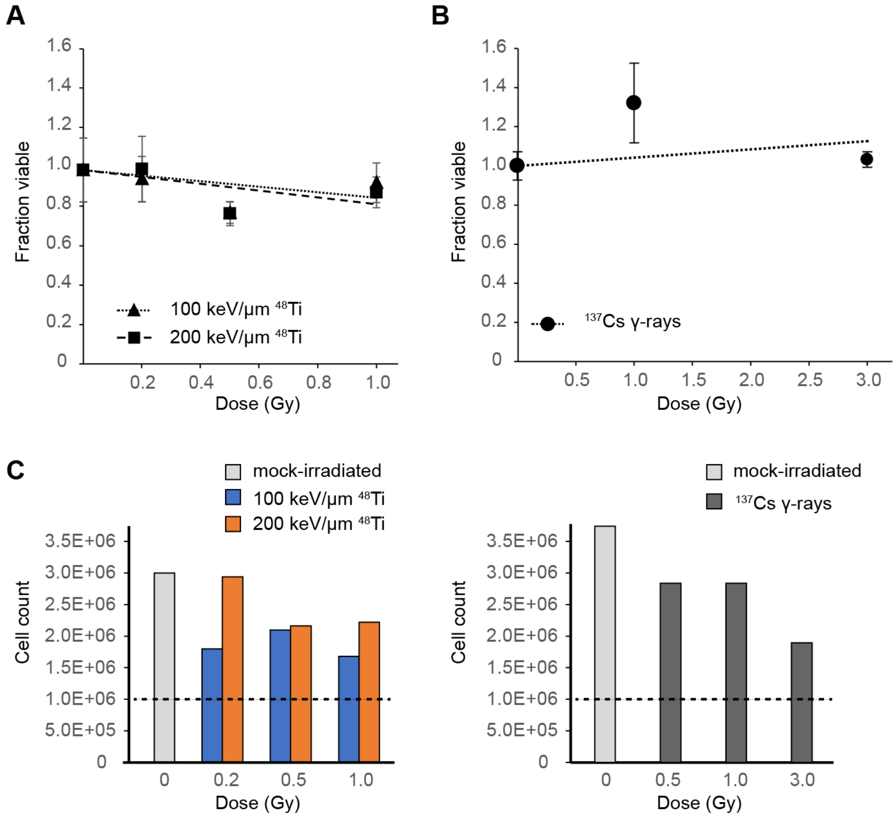

Figure S3. Effect of ^{48}Ti irradiation (LET = 108 keV/µm) on response to Cas9/sgRNA challenge. Duplicate cultures were irradiated at indicated doses, challenged by nucleofection with a Cas9/sgRNA expression vector, and DNA was analysed by SYBR-green qPCR using primers P3 and P4 (Materials and Methods), or with primers P5 and P6, specific to an unaffected region of ALK, as an external standard. Plots show ΔCt from irradiated and non-irradiated control cells, normalized to the external standard. Significance was evaluated by ANOVA followed by 2-sided Dunnett t-tests *, $P < 0.05$.

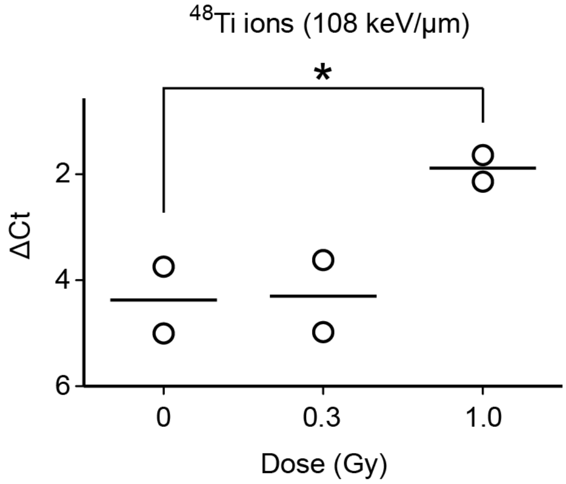

Source data for Fig. S1, Panel D. Gel was photographed, printed on thermal paper, and the printout was scanned to create the image shown. To prepare Fig. S1, Panel D, image was reflected horizontally, grayscale values were inverted, levels were adjusted uniformly to map input greyscale levels 0-203 to output levels 0 to 255, and irrelevant lanes (4,5,6,7,8,9) were cropped. Lane numbers shown correspond to lane numbers in Fig. S1, Panel D.

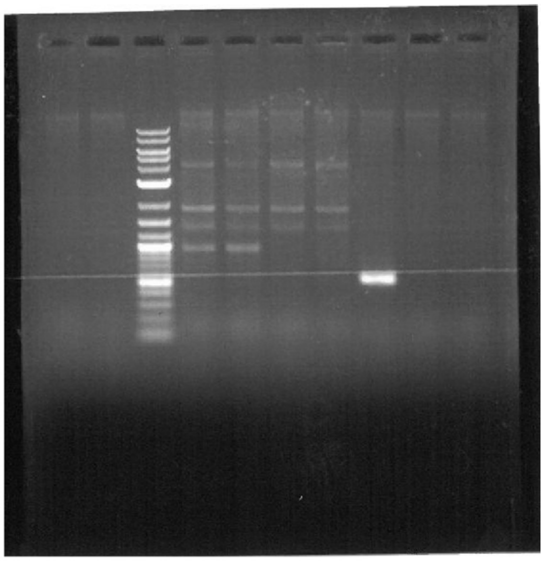

lane: 9 8 M 7 6 5 4 3 2 1
